# Potential Embolic Sources Differ in Patients With Embolic Stroke of Undetermined Source According to Age: A 15-Year Study

**DOI:** 10.3389/fneur.2022.860827

**Published:** 2022-05-17

**Authors:** Yan Hou, Ahmed Elmashad, Ilene Staff, Mark Alberts, Amre Nouh

**Affiliations:** ^1^Department of Neurology, Hartford Hospital, Hartford, CT, United States; ^2^Department of Neurology, University of Connecticut, Farmington, CT, United States; ^3^Department of Research, Hartford Hospital, Hartford, CT, United States

**Keywords:** ESUS, potential embolic source, PFO, ischemic stroke in young adults, AFib

## Abstract

**Introduction:**

Understanding the potential embolic source in young patients with ESUS may improve the diagnosis and treatment of such patients.

**Hypothesis:**

Potential embolic sources (PES) differ in young vs. older patients with ESUS, and, therefore, not all patients with ESUS have the same risk profile for stroke recurrence.

**Methods:**

Young patients (age 18-49) with ESUS, who were admitted to our stroke center from 2006 to 2019, were identified retrospectively and matched with next consecutive older patients (age 50–99) with ESUS by admission date. PES were categorized as atrial cardiopathy, AFib diagnosed during follow-up, left ventricular disease (LVD), cardiac valvular disease (CVD), PFO or atrial septal aneurysm (ASA), and arterial disease. Patients, who had cancer or thrombophilia, were excluded. The type and number of PES and stroke recurrence rates were determined and compared between young and older patients.

**Results:**

In young patients (55.3% women, median age 39 years), the most common PES was PFO/ASA, and the rate of other PES was low (2–7%). Half of the young patients (54.1%) had a single PES, only 10% had multiple PES, and 35.3% of young patients did not have any PES identified. In older patients (41.7% women, median age 74 years), the 3 most common PES were atrial cardiopathy (38.1%), LVD (35.7%), and arterial disease (23.8%). Nearly half of older patients (42.9%) had multiple PES. The rate of stroke recurrence tended to be lower in young patients as compared to older patients (4.9 vs. 11.4%, *p* = 0.29). During a median follow-up of 3 years, only 3 young patients (4.9%) had a recurrent stroke, and two of them had unclosed PFO. There were no recurrent strokes among young patients with no PES identified.

**Conclusions:**

It was noted that PES differ in patients with ESUS according to age and differences in recurrence. PFO is the only common PES in young patients with ESUS. Future studies prospectively evaluating PES in both age groups are needed.

## Introduction

The concept of embolic stroke of undetermined source (ESUS) was introduced by the cryptogenic stroke/ESUS international working group in 2014 (1). In patients with ESUS, the causal etiology might not be identified, but there are many potential embolic sources, also referred to as potential embolic sources (PES) (2, 3). These underlying PES are heterogeneous, but it is hypothesized that emboli are either cardiogenic, arteriogenic, or paradoxical from the veins. At present, the main challenge in ESUS remains how to decide on optimal secondary stroke prevention in the absence of a known stroke mechanism. The efficacy of antithrombotic agents for secondary prevention in ESUS was tested in the NAVIGATE ESUS and RE-SPECT ESUS trials. Results showed that anticoagulation was not superior to aspirin for the prevention of stroke in patients with ESUS (4, 5). Ischemic stroke in young adults is usually defined clinically by an age threshold of <50 at stroke occurrence, based on different risk factors and clinical characteristics between young and older patients with stroke (6). Approximately 15–20% of patients with ESUS are young patients (7, 8). However, previous RCTs and studies included mainly older patients at a mean age of around 65 years (2–5). There is limited information about PES and the associated risk of recurrent stroke in young patients with ESUS. In this retrospective study, we evaluated the rate and overlap degree of previously reported PES and assessed the rate of stroke recurrence per PES in young patients with ESUS and compared them with older patients with ESUS.

## Materials and Methods

We retrospectively reviewed young patients (age 18–49) with ESUS who were admitted and followed at Hartford Hospital from 2006 to 2019. Each young patient was matched with the next consecutive older patient (age 50–99) with ESUS by admission date within the same period.

The ESUS was defined as non-lacunar brain infarct in the absence of extracranial or intracranial atherosclerosis causing ≥50% luminal stenosis in arteries supplying the area of infarcts, established major cardioembolic source, other specific cause (e.g., vasculitis/vasculopathy, dissection, vasospasm, or thrombophilia) according to the criteria proposed by the Cryptogenic Stroke/ESUS International Working Group and previous studies (1). The minimal standard evaluation was required for the diagnosis of ESUS, which included transthoracic or transesophageal echocardiography, CTA/MRA of head and neck, 12-lead EKG, at least 24 hours of continuous heart rhythm monitoring, and screening for inherited thrombophilia in patients younger than 50-year-old (1, 9). We did not include cancer as PES as hypercoagulability and embolism are both possible mechanisms of cancer-related stroke.

According to previous studies, we used the definitions of PES described in Table 1: categorized as atrial cardiopathy, AFib diagnosed during follow-up, left ventricular disease (LVD), cardiac valvular disease (CVD), PFO and/or Atrial septal aneurysm (ASA), and arterial disease (1–3). In a single patient, there may be none, single, or multiple PES. Atrial cardiopathy was defined as left atrial (LA) volume index >34 ml/m^2^ according to American Society of Echocardiography guidelines (10). AFib was diagnosed by EKG, 30-day or implanted heart rhythm monitor during follow-up.

**Table 1:** Definition of potential embolic sources (PES).

**Atrial cardiopathy**	**Left atrium dilation on echo^**a**^**
Afib/Aflutter at follow-up	Atrial Fib/flutter, detected by 30 day or implanted event monitor or by any EKG performed after discharge
Left ventricular dysfunction	heart failure history, or LV ejection fraction was <35%^b^, LV hypertrophy, moderate diastolic dysfunction, global or regional wall motion abnormality
Cardiac valve disease	bioprosthetic valve, mitral valve prolapse, moderate to severe annular calcification, moderate stenosis or regurgitation of mitral or aortic valve
PFO	PFO and/or ASA detected on Echo
Arterial disease	ipsilateral atherosclerotic plaque causing luminal stenosis of <50% or complex aortic arch atherosclerotic plaque^c^

^a^*Left atrial dilation was defined according to Echo lab criteria based on LA volume index >34 ml/m^2^*.

*^b^Patients with LVEF ≤ 25% was excluded*.

*^c^Complex aortic plaque defined as mobile, ulcerated, and/or size ≥4 mm on CTA or TEE*.

Baseline and clinical stroke characteristics were compared between young and older patients using proportions for categorical variables and medians with interquartile range for continuous variables. The rate of different types of PES and numbers of PES were determined and compared between young and older patients with ESUS. After discharge, patients were followed up at Hartford Healthcare for at least one year with the available medical record. Patients who did not follow with Hartford Healthcare for at least one year were considered as lost to follow-up and were not included in the study. The recurrent ischemic stroke was diagnosed by repeated brain MRI or head CT during follow-up. The rate of stroke recurrence during follow-up was assessed by different types of PES, numbers of PES, and comparison with young and older patients with ESUS. All comparisons were performed using the Pearson Chi-square test with SPSS and *p* < 0.05 as the significance level.

## Results

A total of 85 young patients with ESUS (55.3% women, median age 39 years) were identified and matched with 84 older patients (41.7% women, median age 74 years). The baseline and clinical characteristics of patients are summarized in Table 2. Young patients with ESUS tended to be women and ethnic minorities as compared to older patients.

**Table 2:** Baseline and clinical characteristics.

	**Baseline and clinical characteristics**		
	**Young ESUS N = 85**	**Older ESUS N = 84**	***P***
Age	39 (32–44)	74 (63−81)	<0.001
Female	55.3% (47/85)	41.7% (35/84)	0.08
Caucasian	62.4% (53/85)	77.4% (65/84)	0.07
Afr-Am	12.9% (11/85)	4.8% (4/84)	
Other race	24.7% (21/85)	17.9% (15/84)	
Hypertension	23.5% (20/85)	81.0% (68/84)	<0.001
Hyperlipidemia	24.7% (21/85)	61.9% (52/84)	<0.001
Diabetes	17.6% (15/85)	32.1% (27/84)	0.03
Active Smoking	20.0% (17/85)	14.1% (12/84)	0.33
NIHSS (0-5)	65.9% (54/82)	62.0% (49/79)	0.88
NIHSS (6–9)	14.6% (12/82)	16.5% (13/79)	
NIHSS (≥10)	19.5% (16/82)	21.5% (17/79)	
LVO	22.4% (19/85)	17.9% (15/84)	0.63
Thrombectomy	14.1% (12/85)	6.0% (5/84)	0.08
Discharge home	71.6% (58/81)	48.0% (36/75)	0.003
Discharge rehab	28.4% (23/81)	52.0% (39/75)	
Antiplatelet	87.8% (72/82)	79.8% (67/84)	0.16
Anticoagulation	12.2% (10/82)	20.2 (17/84)	
Recurrence after 1 year	2.6% (2/77)	6.3% (5/79)	0.26
Recurrence during follow-up	4.1% (3/74)	11.4% (9/79)	0.29

*Data are given as a percentage (number). Age is presented as median (interquartile range)*.

As expected, a lower proportion of young patients with ESUS had hypertension, hyperlipidemia, and diabetes, although both young and older patients with ESUS had a similar rate of active smoking. The initial NIHSS at admission as well as the rate of large vessel occlusion were similar in young and older patients with ESUS, with young patients tending to receive mechanical thrombectomy. Young patients were more likely to be discharged home compared to older patients. Most young (87.8%) and older (79.8%) patients with ESUS were treated with antiplatelet agents with lower rates of anticoagulation among younger patients. Young patients with ESUS tended to have a lower rate of recurrent embolic stroke during follow-up as compared to older patients.

The most common PES in young patients with ESUS was PFO, with or without an atrial septal defect (ASA) (42.4%). Other types of PES were only identified in 2–7% of young patients with ESUS (Table 3). About half of young patients (54.1%) with ESUS were found to have a single PES, only 10% had multiple PES and about one-third (35.3%) did not have any PES identified (Table 3). In older patients with ESUS, the 3 most common PES were atrial cardiopathy (38.1%), LVD (35.7%), and arterial disease (23.8%). wDuring follow up, AFib was detected in 17.9% of older patients (about 32% of older patients with ESUS had a 30-day monitor and 11% had implanted monitors) as compared to in 2.4% of young patients with ESUS (~20% of young patients with ESUS had a 30-day monitor and 5% received implanted monitor). As compared to young patients, at least one PES was identified in 78.6% of older patients with ESUS. Nearly half (42.9%) of older patients had multiple PES (Table 3).

**Table 3:** Rate of PES by types and numbers.

	**Young ESUS *N* = 85**	**Old ESUS *N* = 84**	***P***
**Individual PES**
Atrial cardiopathy	2.4% (2)	38.1% (32)	<0.001
Afib	2.4% (2)	17.9% (15)	0.001
Left ventricular dysfunction	5.9% (5)	35.7% (30)	<0.001
Cardiac valve disease	5.9% (5)	16.7% (14)	0.026
PFO/ASA	42.4% (36)	9.5% (8)	<0.001
Arterial disease	7.1% (6)	23.8% (20)	0.003
**Number of PES**			
NO PES	35.3% (30)	21.4% (18)	<0.001
Single PES	54.1% (46)	35.7% (30)	
Multiple PES	10.6% (9)	42.9% (36)	

*Data are given as percentages (numbers)*.

The rate of recurrent embolic stroke by PES is summarized in Table 4. During a median follow-up of 3 years, 13% of young patients with ESUS and 6% of older patients with ESUS were lost follow-up. About one out of 10 (11.4%) older patients with ESUS had a recurrent stroke. Older patients who were found to have A-fib during follow-up tended to have the highest rate of stroke recurrence (26.7%). Older patients who had multiple PES had a rate of 15.8% stroke recurrence compared to those who had single or no PES at 6.7% and 5.6%, respectively. Only one out of 25 young patients with ESUS (4.1%) had a recurrent stroke. Among the three young patients who had a recurrent stroke, two patients had PFO without closure and one patient had cardiac valve disease. There were no recurrent strokes among young patients with no PES identified.

**Table 4:** Recurrent embolic stroke by type and number of PES.

	**Stroke recurrence**	
**PES**	**Young ESUS**	**Old ESUS**
Atrial cardiopathy	0/2	18.8% (6/32)
Afib at F/U	0/2	26.7% (4/15)
Left ventricular dysfunction	0/5	13.3% (4/30)
Cardiac valve disease	20.0% (1/5)	14.2% (2/14)
PFO/ASA	5.6% (2/36)	12.5% (1/8)
Arterial disease	0/6	10% (2/20)
No PES	0/30	5.6% (1/18)
Single PES	6.5% (3/46)	6.7% (2/30)
Multiple PES	0/7	15.8% (6/38)
Total	4.1% (3/74)	11.4% (9/79)

*Data are given as a percentage (number)*.

## Discussion

In this observational study, PFO and/or ASA were the only common PES in young patients with ESUS and found in nearly half (42%) of young patients. In contrast, PFO and/or ASA were detected in less than 10% of older patients, albeit many older patients who usually underwent transthoracic echocardiography without bubble study. Other PES, such as atrial cardiopathy, LVD, arterial disease, AFib, and cardiac valve disease, were frequently identified in about 15–40% of older patients, but they were uncommonly detected in less than 10% of young patients. Overall, young patients with ESUS had a lower burden and less overlap of PES than older patients. Half (54%) of young patients had single PES, and one-third of young patients did not have any PES identified, whereas nearly half of older patients had two or three PES. Our findings are consistent with a recent study that identified three main clusters of patients with ESUS according to clinical characteristics and cerebrovascular risk factors. Those clusters were correlated to the potential arteriogenic, cardiogenic, and paradoxical source of embolic stroke. One cluster was associated with PFO in patients at young ages (11).

According to a systemic review including most studies of older patients, ESUS is associated with a recurrent stroke rate of 4.5% per year (8). Among the young patients registered to the Helsinki Young Stroke Registry, young patients with ESUS had a relatively low cumulative risk of recurrent stroke that is about 1.95% annually over a median follow-up time of 10 years (7). Again, our study showed young patients with ESUS tended to have a lower rate of stroke recurrence as compared with older patients with ESUS. Only three young patients had a recurrent stroke, and two of them had PFO without closure. In recent years, emerging evidence indicates that paradoxical embolism through PFO is a potential causal mechanism of ESUS (12). PFO with large shunting and/or ASA was associated with a high risk for recurrent stroke (12, 13). Two recent RCTs found that percutaneous closure of PFO in appropriately selected patients decreases the risk of recurrent stroke in young patients with ESUS (14, 15). Only one patient with cardiac valve disease had a recurrent stroke for other PES. The rate of other PES-associated stroke recurrence is difficult to be interpreted in our study because of their low detection rate in young patients with ESUS. As compared to young patients, older patients who were found to have A-fib during follow-up tended to have the highest rate of stroke recurrence (26.7%). And all other PES-associated stroke recurrence rate was 10–20% in older patients with ESUS. However, given half of older patients had multiple PES, it is difficult to determine the PES that are potentially implicated in each patient. Previous studies found that older patients with multiple PES were not at increased risk of recurrent stroke as compared to patients who had single PES (2, 3). The finding may indicate high-risk PES, but not multiple low-risk PES, play the key role in stroke recurrence. Young patients with ESUS had less overlap of PES; our study showed that among 10% of young patients who had two PES identified, none of them had a recurrent stroke.

The low rate of PES in young patients with ESUS is partly explained by the low rate of traditional cerebrovascular risk factors. In addition, according to recommendations from the international ESUS working group, only minimal standard evaluation is required to rule out established etiology, other diagnostic tests such as malignancy screening, hypercoagulable testing, 30-day or implanted rhythm monitors, TEE, and special MRIs may be considered on a case-by-case basis (1). PES detection rate may be higher if all the young patients underwent TEE, prolonged cardiac monitoring, and more advanced diagnostic workup. It is important to note, however, that occult AFib was a rare finding in younger patients with ESUS in our study, and of low yield. High-resolution MRI had higher sensitivity and specificity in detecting vessel wall abnormality such as atheroma and dissection (16, 17). If high-resolution MRI can be used in routine practice, it may help to identify more PES in young patients. Furthermore, young stroke patients have unique risk factors such as migraines, the use of oral contraceptive pills, or illicit drugs (18–20). Mechanisms other than embolisms such as hypercoagulability, vasospasm, and *in-situ* thrombosis may be the cause of cryptogenic stroke in young patients. Cancer, especially active cancer is considered a risk factor for ischemic stroke in young adults (21, 22). The hypercoagulable state is the most important mechanism of malignancy-associated ischemic stroke and other potential mechanisms include marantic endocarditis, the effect of chemotherapy or radiotherapy, accelerated atherosclerosis, and intravascular disease (Lymphoma) (22). Inherited thrombophilia is a group of disorders including factor V Leiden or Prothrombin mutation, deficiency of Antithrombin, protein C and protein S, and hyperhomocysteinemia caused by mutations in methylenetetrahydrofolate reductase (23, 24). Studies have shown an inconsistent or weak association between inherited thrombophilia and ischemic stroke (23–25), although a moderately stronger risk was reported in a subgroup of younger patients (25, 26). Our study focused on ESUS with embolism as a likely cause of stroke and we did not include patients with malignancy or inherited thrombophilia, in which a hypercoagulable state is a potential mechanism. However, malignancy and thrombophilia are important risk factors for cryptogenic stroke in young adults and they can cause imaging patterns similar as embolic stroke. Of note, our study shows PES was not identified in one-third of young patients with ESUS, and none of those patients had a recurrent stroke. Although the causal mechanism remains unknown, this finding is reassuring that stroke recurrence was rare in these young patients with ESUS without PES identified.

The findings of our study suggest young patients differ from older patients with ESUS. Although the two groups have similar stroke severity and rate of large vessel occlusion, they are different regarding risk factors, possible embolic source, and stroke recurrence. Current RCTs testing antithrombotics for stroke prevention in ESUS mainly focused on older patients and the finding would not be well-applicable to young patients (5, 6). Severe atrial cardiopathy was identified as an independent risk factor in the absence of AFib in patients with ESUS (27). However, due to the low prevalence of atrial cardiopathy in younger patients, the ongoing ARCADIA (Atrial Cardiopathy and Antithrombotic Drugs in Prevention after Cryptogenic Stroke) trial only enrolls patients with ESUS older than 45 years (28). PFO is the only common PES in young patients with ESUS. Given the low rate of other PES, their roles in stroke recurrence are less significant in young patients with ESUS. Future studies in young patients with ESUS should focus on PFO-related stroke to better define features of high-risk PFO and identify appropriate patients to benefit from PFO closure as it will further lower stroke recurrence.

### Strengths and Limitations

Our study is the first study to assess PES and its associated rate of stroke recurrence in young patients with ESUS over 15 years. However, due to a single-center design, we were unable to estimate the risk of recurrent stroke by PES given the relatively small number of patients. One common limitation of all PES studies is that the rate and overlap of PES are usually underestimated. PES in our study was defined according to criteria used in NAVIGATE ESUS study and reviewed by an international ESUS working group (1, 2). Many recently identified PES such as new biomarkers of atrial cardiopathy, including NT-proBNP and ECG indexes (28–30), pulmonary arteriovenous fistula, and carotid web, were not included in the study (31, 32). Another common limitation in PES studies is that the criteria also likely include many low-risk PES. Those PES are usually associated with a very low rate of stroke and are often likely incidental without clinical significance. In the future, a prospective multi-center study better defined PES, with a sufficient sample size and completion of extensive diagnostic tests, is needed to assess the PES burden and associated risk of stroke recurrence in young patients with ESUS.

In conclusion, PES differs in patients with ESUS according to age. PFO is the only common PES in young patients with ESUS. Young patients have less burden and overlap with other PES that are commonly identified in older patients. Future research should continue to identify high-risk PES specific to young patients and verify features of high-risk PFO.

## Data Availability Statement

The raw data supporting the conclusions of this article will be made available by the authors, without undue reservation.

## Ethics Statement

The studies involving human participants were reviewed and approved by IRB of Research Center at Hartford Hospital. Written informed consent for participation was not required for this study in accordance with the National Legislation and the Institutional Requirements.

## Author Contributions

YH: study design, data collection, and manuscript writing and revision. AN: study design and manuscript revision. MA: manuscript revision. IS: data analysis and manuscript revision. AE: data collection. All authors contributed to the article and approved the submitted version.

## Conflict of Interest

The authors declare that the research was conducted in the absence of any commercial or financial relationships that could be construed as a potential conflict of interest.

## Publisher's Note

All claims expressed in this article are solely those of the authors and do not necessarily represent those of their affiliated organizations, or those of the publisher, the editors and the reviewers. Any product that may be evaluated in this article, or claim that may be made by its manufacturer, is not guaranteed or endorsed by the publisher.
